# The effect of intraoperative dexamethasone on glycaemic responses in people with diabetes mellitus: a preplanned analysis of the Perioperative ADministration of Dexamethasone and Infection trial

**DOI:** 10.1016/j.bja.2026.05.009

**Published:** 2026-06-16

**Authors:** Leon A. Bach, Catherine Martin, Richard J. MacIsaac, Paul S. Myles, Mohammad Asghari-Jafarabadi, Edmond O'Loughlin, David Story, Kwok Ho, Jaspreet Sidhu, Tomas B. Corcoran, For the PADDI investigators

**Affiliations:** 1 Department of Endocrinology and Diabetes, The Alfred, Melbourne, VIC, Australia; 2 School of Translational Medicine, Monash University, Melbourne, VIC, Australia; 3 School of Public Health and Preventive Medicine, Monash University, Melbourne, VIC, Australia; 4 Department of Endocrinology & Diabetes, St Vincent’s Hospital Melbourne, Fitzroy, VIC, Australia; 5 Department of Medicine, St Vincent's Hospital Melbourne and University of Melbourne, Fitzroy, VIC, Australia; 6 Australian Centre for Accelerating Diabetes Innovations, School of Medicine, University of Melbourne, Parkville, VIC, Australia; 7 Department of Anaesthesiology and Perioperative Medicine, Alfred Hospital and Monash University, Melbourne, VIC, Australia; 8 Biostatistics Unit, School of Public Health and Preventive Medicine, Monash University, Melbourne, VIC, Australia; 9 Department of Psychiatry, School of Clinical Sciences, Monash University, Clayton, VIC, Australia; 10 Cabrini Research, Cabrini Health, Malvern, VIC, Australia; 11 Department of Anaesthesia, Pain and Perioperative Medicine, Fiona Stanley Hospital, Murdoch, VIC, Australia; 12 Department of Critical Care, University of Melbourne, Parkville, VIC, Australia; 13 Department of Anaesthesia and Intensive Care, Prince of Wales Hospital and The Chinese University of Hong Kong, Hong Kong SAR, China; 14 Department of Epidemiology and Preventive Medicine, School of Public Health and Preventive Medicine, Monash University, Melbourne, VIC, Australia; 15 Department of Anaesthesia and Pain Medicine, Royal Perth Hospital, Perth, WA, Australia; 16 School of Medicine and Pharmacology University of Western Australia, Perth, WA, Australia

**Keywords:** anaesthesia, diabetes, glucose, intraoperative dexamethasone, surgical-site infection

## Abstract

**Background:**

The Perioperative ADministration of Dexamethasone and Infection (PADDI) randomised placebo-controlled trial showed that a single 8 mg dose of intraoperative dexamethasone had no effect on surgical-site infection within 30 days after nonurgent, noncardiac surgery. Whether dexamethasone increases blood glucose and adverse outcomes in people with diabetes mellitus remains unclear.

**Methods:**

In this preplanned sub-study, 1130 PADDI participants (mean age 65.2 y: 40.7% female) with diabetes mellitus and glycated haemoglobin <9.0% were included in this ‘as treated’ analysis. The primary outcome was the maximum recorded perioperative blood glucose concentration (mM) within 24 h of anaesthesia induction.

**Results:**

The maximum perioperative blood glucose level was 12.5 mM (median, IQR 10.6–15.0) in 570 participants receiving dexamethasone, compared with 10.3 mM (8.8–12.4) in 560 participants receiving placebo (estimated difference 1.86 mM (95% CI 1.50–2.22), *P*<0.001). There was no evidence of increased risk of surgical-site infection (dexamethasone 11.1% *vs* placebo 14.4%, risk ratio [RR], 0.74; 95% confidence interval [CI], 0.54–1.01). Age, sex, BMI and HbA1c were not associated with maximum perioperative glucose levels or the incidence of surgical-site infections between the dexamethasone and placebo groups. The requirement for additional insulin and the number of hypoglycaemic episodes did not differ between groups.

**Conclusion:**

In people with diabetes mellitus, dexamethasone was associated with a modest increase in perioperative blood glucose but not with increased surgical-site infection. These findings support the use of dexamethasone to prevent postoperative nausea and vomiting in people with diabetes mellitus.

**Editor’s key points**

• In the PADDI RCT, intraoperative dexamethasone did not increase surgical-site infection after nonurgent noncardiac surgery.
• The authors re-examined PADDI data to assess the impact of intraoperative dexamethasone on glycaemic control in people with diabetes mellitus.
• In people with diabetes mellitus, dexamethasone increased maximum perioperative blood glucose concentrations compared with placebo.
• Despite higher perioperative glucose concentrations, dexamethasone was not associated with increased surgical-site infection, insulin requirements, or hypoglycaemic episodes.
• These findings support the continued use of dexamethasone for postoperative nausea and vomiting prophylaxis in surgical patients with diabetes mellitus.

Hyperglycaemia is a stress response to surgery comprising complex neuroendocrine and inflammatory components.^1^ It is characterised by insulin resistance, excessive hepatic gluconeogenesis and glycogenolysis, peripheral lipolysis, and increased whole-body glucose uptake and oxidative glucose metabolism. The clinical relevance, potential harm and optimal approach to manage perioperative hyperglycaemia remain topical but studies are limited.^2^^,^^3^

Dexamethasone is commonly used perioperatively for several indications, particularly prevention and treatment of postoperative nausea and vomiting, for which it is highly effective.^4^ Given the effects of dexamethasone on immune function, there were concerns that dexamethasone may increase the risk of surgical site infection (SSI). However, a number of studies, including the large, randomised, multicentre Perioperative ADministration of Dexamethasone and Infection (PADDI) study, have allayed these fears, with the latter showing that 8 mg of dexamethasone was non-inferior to placebo with respect to SSI within 30 days.^5^ Furthermore, dexamethasone is a glucocorticoid that may increase blood glucose, particularly in those with impaired blood glucose tolerance or diabetes mellitus. Previous studies have shown that perioperative hyperglycaemia is associated with increased SSI, especially in the context of cardiac surgery, and that intensive insulin treatment decreases this risk.^6^, ^7^, ^8^ However, a recent retrospective study did not show an effect of perioperative glucose on SSI in patients with diabetes mellitus undergoing cardiac surgery in the context of improved infection control measures and glucose management.^9^

In the PADDI study, which included 1157 participants with diabetes mellitus,^5^ dexamethasone did not increase the risk of SSI. In the entire cohort, the median postoperative blood glucose level increment was 3.6 mM (interquartile range [IQR], 2.5–4.9) and 2.4 mM (1.4–3.6) in the dexamethasone and placebo groups, respectively. However, that study did not include a detailed analysis of the relationships between dexamethasone, postoperative glucose and SSI in patients with diabetes mellitus. The primary aim of the current preplanned analysis was therefore to determine the effect of a single dose of intraoperative dexamethasone on perioperative glycaemic responses, as determined by the maximum perioperative blood glucose concentration from the time of anaesthesia induction to day 1 after surgery. Secondary aims included the effects of dexamethasone on other measures of the glucose response and SSI risk.

## Methods

### Study design

PADDI was a pragmatic, international, multicentre, randomised, placebo-controlled, triple-blind, noninferiority trial of 8 mg intravenous dexamethasone after induction of general anaesthesia and before surgical incision.^5^ Written informed consent was obtained from all participants. Alfred Health oversaw the study, which was approved by its Ethics Committee (HREC/15/Alfred/22 [local reference: 334/15]) and relevant committees at all participating institutions. This glycaemic control sub-study was preplanned in the original PADDI protocol (version 2.1),^5^ and did not require separate patient consent. All data collected for this study were prespecified.

### Participant selection and trial procedures

In the PADDI trial, patients were recruited at 55 sites in 4 countries between March 2016 and July 2019. A total of 8880 patients were randomised and 8725 included in the modified intention-to-treat population. Trial details and its findings are described elsewhere.^5^^,^^10^ Briefly, adult patients undergoing elective or expedited noncardiac, non-obstetric surgery under general anaesthesia with an expected hospital stay of at least one night were included. Randomisation was stratified by diabetes mellitus status, with diabetes mellitus defined by ‘self-declared’ diabetes mellitus or HbA1c >6.4% and preoperative glucose >11.1 mM (mmol l^−1^). 1157 patients with diabetes mellitus were included in the PADDI trial. Poorly controlled diabetes mellitus, defined by HbA1c >9.0%, was an exclusion criterion. Baseline demographic data (age, sex, weight, BMI, diabetes mellitus type, duration, treatment and complications) were obtained. Patients were randomised 1:1 to receive a single intravenous dose of 8 mg dexamethasone or placebo within 5 min after anaesthesia induction. Blood glucose was measured at induction either in the laboratory or using point-of-care devices. HbA1c was measured within 30 days before surgery or at induction. Surgical and anaesthesia details were collected. The maximum intraoperative glucose level was recorded. After surgery, maximum blood glucose in the postanaesthesia care unit (PACU) was recorded, and levels 8–12 h after surgery and on day 1 after surgery. Intraoperative insulin administration (single dose, multiple dose or infusion) and insulin administration outside of usual care within the first 24 h was recorded. Perioperative diabetes management was delivered according to local institutional protocols or, if applicable, under the guidance of the study protocol. SSI within 30 days was assessed using Centers for Disease Control and Prevention definitions.^11^

### Primary outcome

The primary outcome was the maximum recorded perioperative blood glucose concentration from the time of anaesthesia induction to day 1 after surgery (24 h after surgical incision).

### Secondary outcomes

Prespecified secondary outcomes were: 1) The maximum intraoperative and PACU blood glucose concentrations; 2) Blood glucose values 8–12 h after the study drug administration and on day one; 3) The maximum change in blood glucose from induction to the maximum glucose during (a) the perioperative period, (b) 8–12 h after the study drug administration; 4) Proportion of glucose <4.0 mM during the perioperative period; 5) Requirement for supplementary insulin during the perioperative period; and 6) SSI within 30 days.

### Statistical analysis

The statistical analysis plan was finalised and published online on November 2, 2023 (https://www.anzca.edu.au/getContentAsset/c90106a4-64ad-4532-82de-f07a9932c758/80feb437-d24d-46b8-a858-4a2a28b9b970/PADDI-glycaemia-SAP-2Nov-FINAL-to-publish.pdf?language=en&view=1) before the commencement of analyses. Analysis and reporting follow STrengthening the Reporting of OBservational studies in Epidemiology (STROBE) guidelines.^12^

Participants with diabetes mellitus from the PADDI modified intention-to-treat population were included in this analysis. They were excluded if they had more than one dose of dexamethasone during surgery, had non-study dexamethasone within 24 h or had missing information regarding non-study dexamethasone.

Participants were then categorised into those who received a single dose of dexamethasone during surgery and those who did not. To account for the non-randomised nature of this ‘as treated’ analysis, all analyses were adjusted for the following potential confounders: previous diabetes mellitus complications and non-insulin injectables (because of the imbalance between the groups), and type of dominant surgery and preoperative HbA1c (considered as important potential confounders).

The primary outcome and other skewed data were analysed using quantile regression with robust standard errors, resulting in a difference in medians and 95% CI. Binary data were analysed by regression, resulting in an RR estimate. For non-convergence issues, Poisson regression with robust standard errors was used, resulting in an incidence RR. Ordinal logistic regression, resulting in an odds ratio, was used to analyse the most intensive insulin regimen.

The following subgroups were analysed: age quartiles, sex, BMI categories, surgery duration quartiles, and HbA1c categories. The subgroups were analysed through an interaction test in regression models for the primary endpoint, and forest plots were constructed. The same subgroups were also analysed for SSI.

The relationship between dexamethasone and maximum blood glucose on SSI was examined using binary regression with SSI as the outcome and an interaction term between maximum blood glucose and dexamethasone during surgery, adjusting for confounders mentioned above, resulting in an RR estimate.

A sensitivity analysis used multiple imputation by chained equations for missing glucose measurements that contributed to determining the primary outcome. Continuous measures were imputed through predictive mean matching. Binary variables used logistic imputation with augmentation to address separation. The three-level insulin regimen was imputed with multinomial logit. Models included clinically relevant predictors and missingness indicators to cater for missing at random. All analyses were fitted with mi estimates, combining results using Rubin’s rules. An additional sensitivity intention-to-treat analysis was undertaken on the diabetes mellitus stratum of the original PADDI randomised population.

For all outcomes, two-sided 95% CIs are presented. A nominal 5% significance level was used. Statistical analysis was performed using Stata versions 18 and 19.5 (StataCorp, College Station, Texas, USA).

## Results

### Study characteristics

**Fig 1:**
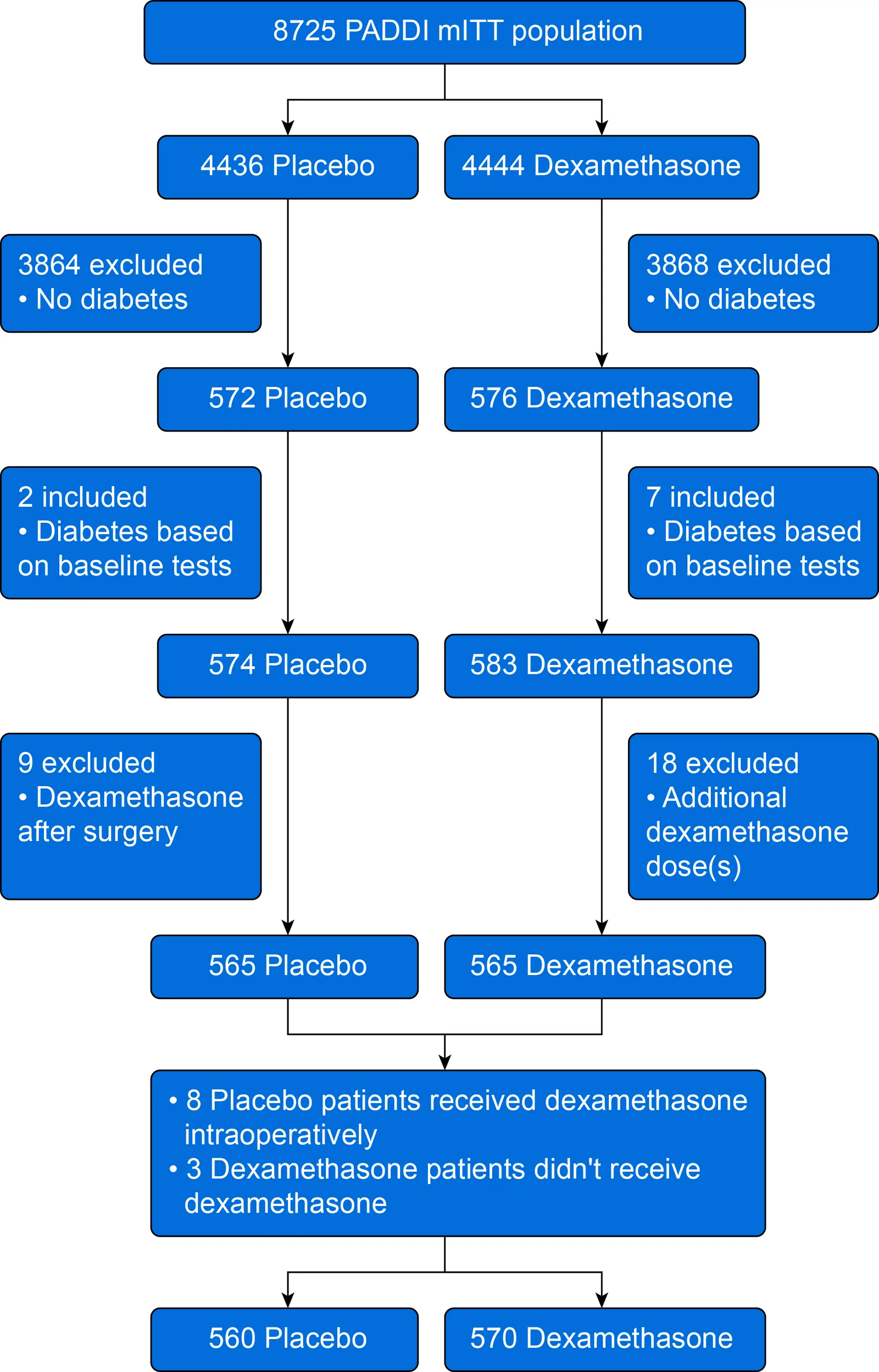
Patient flow diagram.

Of the 8880 participants randomised in the PADDI trial,^5^ 1157 had diabetes mellitus. 574 participants were randomised to placebo, and 583 participants were randomised to receive dexamethasone (Fig. 1). Nine participants in the placebo group were excluded because they received dexamethasone within the first 24 h after surgery and 18 participants in the dexamethasone group were excluded because they received additional doses above the study dose. A further eight participants in the placebo group received one dose of dexamethasone intraoperatively and were transferred to the dexamethasone group in this ‘as treated’ analysis. Conversely, three participants in the dexamethasone group did not receive it and were transferred to the placebo group. Final numbers in the placebo and dexamethasone groups were 560 and 570, respectively, for this ‘as treated’ observational analysis of participants who received only one dose of dexamethasone or placebo (Table 1 and Table S1).

**Table 1:** Baseline characteristics by dexamethasone status in patients with diabetes mellitus. Results are shown as mean (standard deviation) or median (interquartile range).

	Total	Placebo	Dexamethasone
	*n*=1130	*n*=560	*n*=570
**Age (yr, range)**	21–92	25–92	21–90
**Male, *n* (%)**	670 (59.3%)	329 (58.8%)	341 (59.8%)
**Body weight (kg)**	88.7 (23.0)	89.2 (22.2)	88.2 (23.6)
**BMI (kg m^−2^)**	31.5 (7.4)	31.7 (7.1)	31.3 (7.6)
**ASA physical status 3**–**4**	615 (54.4%)	313 (55.9%)	302 (53.0%)
**Type of diabetes mellitus**			
**Type 1**	22 (1.9%)	8 (1.4%)	14 (2.5%)
**Type 2**	1098 (97.2%)	548 (97.9%)	550 (96.5%)
**Other**	10 (0.9%)	4 (0.7%)	6 (1.1%)
**Years since diagnosis**	8.0 (4.0, 15.0)	8.0 (4.0, 14.0)	8.0 (4.0, 15.0)
**Daily insulin, *n* (%)**	209 (18.5%)	103 (18.4%)	106 (18.6%)
**Oral hypoglycaemic agents, *n* (%)**	872 (77.2%)	437 (78.0%)	435 (76.3%)
**Non-insulin injectables, *n* (%)**	31 (2.7%)	10 (1.8%)	21 (3.7%)
**No diabetes mellitus medication, *n* (%)**	206 (18.2%)	101 (18.0%)	105 (18.4%)
**Diabetes mellitus Complications, *n* (%)**	185 (16.5%)	76 (13.6%)	109 (19.4%)
**Retinopathy**	69 (6.1%)	28 (5.0%)	41 (7.2%)
**Neuropathy**	97 (8.6%)	40 (7.1%)	57 (10.0%)
**Nephropathy**	56 (5.0%)	19 (3.4%)	37 (6.5%)
**Current Smoker, *n* (%)**	146 (12.9%)	70 (12.5%)	76 (13.3%)
**Apfel Score**	2.0 (2.0, 3.0)	2.0 (2.0, 3.0)	2.0 (2.0, 3.0)
**Prophylactic antibiotics, *n* (%)**	1112 (98.4%)	553 (98.8%)	559 (98.1%)
**Contaminated wound, *n* (%)**	428 (37.9%)	218 (38.9%)	210 (36.8%)
**Metal/Synthetic material implanted, *n* (%)**	345 (30.5%)	172 (30.7%)	173 (30.4%)
**Duration of surgery (h)**	2.5 (1.8, 3.7)	2.5 (1.8, 3.6)	2.7 (1.8, 3.7)
**HbA1c (%)**	6.7 (6.1, 7.5)	6.7 (6.1, 7.4)	6.8 (6.1, 7.5)
**HbA1c <7.0%, *n* (%)**	651 (57.7%)	315 (56.1%)	336 (60.5%)
**HbA1c 7.0**–**7.9%, *n* (%)**	266 (23.6%)	137 (24.4%)	129 (23.2%)
**HbA1c >7.9%, *n* (%)**	209 (18.5%)	109 (19.4%)	90 (16.2%)
**HbA1c (mM)**	50 (43, 58)	50 (43, 57)	51 (43, 58)
**Blood glucose (mM)**	7.2 (5.9, 9.0)	7.2 (5.8, 8.9)	7.2 (6.0, 9.2)
**CRP (mg L^−1^)**	2.8 (1.0, 5.6)	2.8 (1.0, 5.0)	2.8 (1.1, 6.3)
**Haemoglobin (g L^−1^)**	137.0 (125.0, 147.0)	137.0 (126.0, 147.0)	138.0 (124.0, 148.0)
**Lymphocytes (×10^9^ L^−1^)**	2.0 (1.5, 2.6)	2.0 (1.5, 2.6)	1.9 (1.5, 2.6)
**Neutrophils (×10^9^ L^−1^)**	4.6 (3.6, 5.6)	4.5 (3.6, 5.6)	4.6 (3.6, 5.6)
**Creatinine (μM)**	77.0 (65.0, 95.0)	76.0 (64.0, 91.0)	78.0 (67.0, 97.0)
**Blood glucose on induction (mM)**	6.9 (5.8, 8.3)	6.8 (5.7, 8.2)	7.0 (5.9, 8.5)

Baseline and intraoperative characteristics of the patient groups are shown in Table 1 and Table S1. There were no clinically important differences between groups. Mean age was 65.2 (SD 11.0) years, BMI 31.5 (7.4) kg m^-2^, 97.2% had type 2 diabetes, and median duration of diabetes mellitus was 8.0 (IQR 4.0, 15.0) years. Baseline glucose on induction was 6.9 (5.8, 8.3) mM. Mean HbA1c was 6.7% (6.1, 7.5) and 42.1% of patients had HbA1c levels at or above the general target of 7.0%. Gastrointestinal, orthopaedic and urology/renal surgery were most common.

### Primary outcome: maximum perioperative blood glucose level

The primary outcome, the maximum perioperative blood glucose level (median, [IQR]), was 12.5 mM (10.6–15.0) in the dexamethasone group and 10.3 mM (8.8–12.4) in the placebo group, estimated difference 1.86 mM (95% CI 1.50–2.22), *P*<0.001, Table 2). A sensitivity analysis, where missing values for each component of maximum perioperative blood glucose were imputed, showed a similar result (estimated median difference 1.87 mM (95% CI 1.54–2.20), *P*<0.001, Table 2). An additional intention-to-treat sensitivity analysis of the diabetes mellitus stratum of the original PADDI randomised population also showed a similar result (Table S2).

**Table 2:** Primary and secondary outcomes. ∗Difference in medians; **^†^**RR; ^‡^OR. All analyses were adjusted for prevalence of diabetic complications, surgery type, preoperative HbA1c and use of non-insulin injectables. All glucose measurements are reported as mM. BG, blood glucose; POC, point of care.

	Placebo (*n*=560)	Dexamethasone (*n*=570)	Estimate (95% CI)	*P*-value	Missing (*n*)
**Maximum perioperative blood glucose (mM), median (IQR)∗**	10.3 (8.8, 12.4)	12.5 (10.6, 15.0)	1.86 (1.50, 2.22)	<0.001	0
**Imputed result**			1.87 (1.54, 2.20)	<0.001	
**Maximum intraoperative blood glucose, median (IQR)∗**	8.2 (6.8, 10.2)	9.4 (7.5, 11.3)	0.81 (0.47, 1.15)	<0.001	48
**Maximum blood glucose in PACU, median (IQR)∗**	8.7 (7.1, 10.4)	10.2 (8.6, 12.1)	1.25 (0.93, 1.58)	<0.001	5
**Blood glucose (POC) 8–12 h post dose, median (IQR)∗**	9.0 (7.6, 10.9)	11.4 (9.3, 14.1)	2.25 (1.88, 2.61)	<0.001	11
**Blood glucose day 1, median (IQR)∗**	8.4 (6.9, 10.3)	9.3 (7.7, 11.7)	0.87 (0.52, 1.23)	<0.001	8
**Change from induction to maximum perioperative blood glucose (mM, median (IQR)∗**	3.3 (1.8, 5.1)	5.1 (3.6, 7.1)	1.81 (1.43, 2.19)	<0.001	43
**Change from induction to blood glucose at 8-12 h (mM, median (IQR)∗**	2.0 (0.5, 3.8)	4.2 (2.6, 6.5)	2.19 (1.79, 2.59)	<0.001	52
**BG <4.0 mM during the perioperative period^†^**	5 (0.9%)	4 (0.8%)	0.64 (0.15, 2.71)	0.54	65
**Insulin required outside of usual treatment during the perioperative period^†^**	112 (20.0%)	150 (26.6%)	1.18 (0.91, 1.51)	0.21	2
**Highest dosage type of insulin outside of usual care^‡^**			1.20 (0.89, 1.61)	0.24	0
**None**	448 (80.0%)	420 (73.7%)			
**Single dose**	20 (3.6%)	36 (6.3%)			
**Multiple doses**	12 (2.1%)	32 (5.6%)			
**Infusion**	80 (14.3%)	82 (14.4%)			

### Secondary outcomes

**Fig 2:**
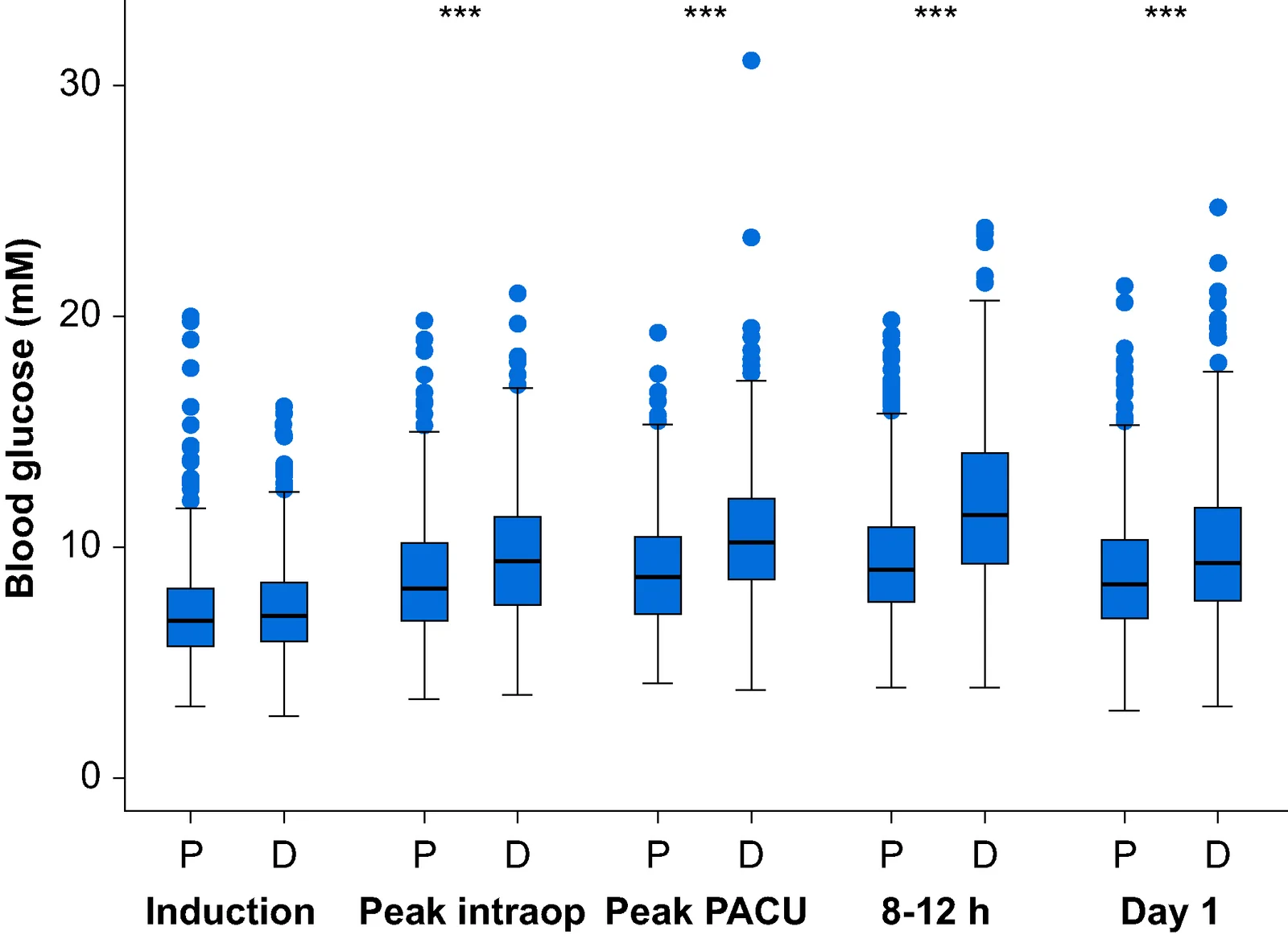
Box- and whisper plot of the time course of maximum blood glucose levels. ∗∗∗ *P*<0.001, dexamethasone *vs* placebo. P, Placebo; D, dexamethasone.

Maximum glucose levels at each individual time point (intraoperative, PACU, 8–12 h after surgery and on day 1 after surgery) were higher in the dexamethasone group, with the maximum effect 8–12 h after surgery (median 11.4 [9.3–14.1] *vs* 9.0 [7.6–10.9] mM, estimated difference 2.25 mM [95% CI 1.88–2.61], *P*<0.001) (Table 2 and Fig. 2). At the time of highest glucose (8–12 h after surgery), 67.4% and 17.7% of maximum values were >10 and >15 mM in the dexamethasone group, respectively, *vs* 33.5% and 5.9% in the placebo group (Table S3). However, ≤8.3% of glucose values were >15 mM in either group at all other time points.

The median increase in blood glucose from induction to the maximum perioperative glucose level was 5.1 mmol.L^-1^ (3.6–7.1) in the dexamethasone group and 3.3 mM (1.8–5.1) in the placebo group (estimated median difference 1.81 mM (95% CI 1.43–2.19), *P*<0.001, Table 2). The increase in glucose from induction to 8–12 h postinduction was 4.2 mM (2.6–6.5) in the dexamethasone group and 2.0 mM (0.5–3.8) in the placebo group (estimated median difference 2.19 mM (95% CI 1.79–2.59), *P*<0.001, Table 2).

**Fig 3:**
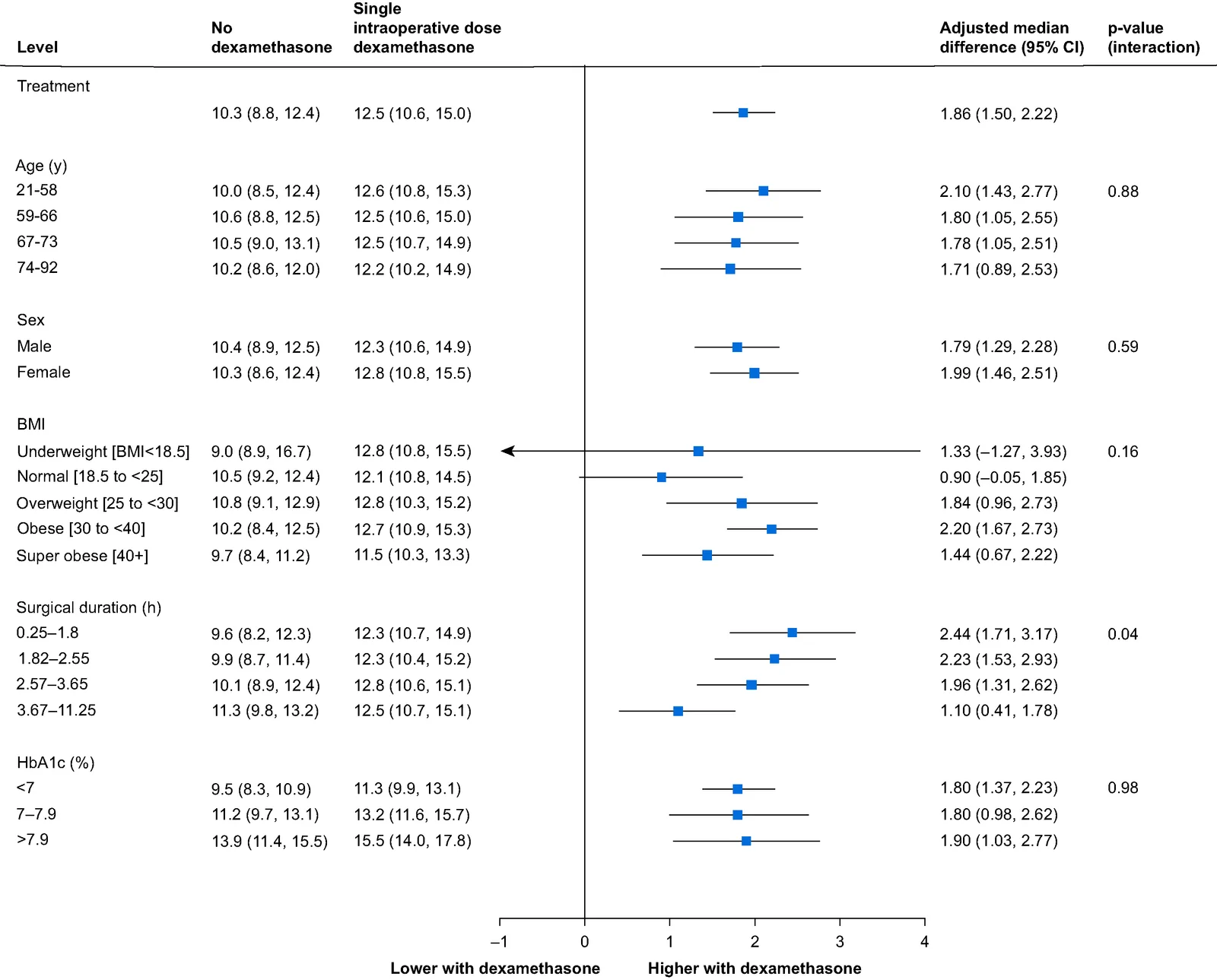
Subgroup analysis - Maximum blood glucose level during the perioperative period. The x-axis depicts adjusted median difference (mM). The widths of the confidence intervals for subgroup analyses were not adjusted for multiplicity and should not be used to infer definitive treatment effects.

Longer surgical duration was associated with a smaller difference in maximum perioperative blood glucose between the dexamethasone and placebo groups (Fig. 3, *P*=0.04). Maximum levels appeared higher with longer operations in the placebo group, whereas there was no difference in the dexamethasone group. In contrast, the difference in maximum perioperative glucose levels between dexamethasone and placebo groups was not associated with age, sex, BMI or HbA1c (Fig. 3). The requirement for insulin in addition to usual treatment (dexamethasone 26.6% *vs* placebo 20.0%, *P*=0.21, Table 2) and the number of hypoglycaemic episodes (blood glucose <4 mM) did not differ between the dexamethasone and placebo groups (dexamethasone 0.8% *vs* placebo 0.9%, *P*=0.54, Table 2).

**Fig 4:**
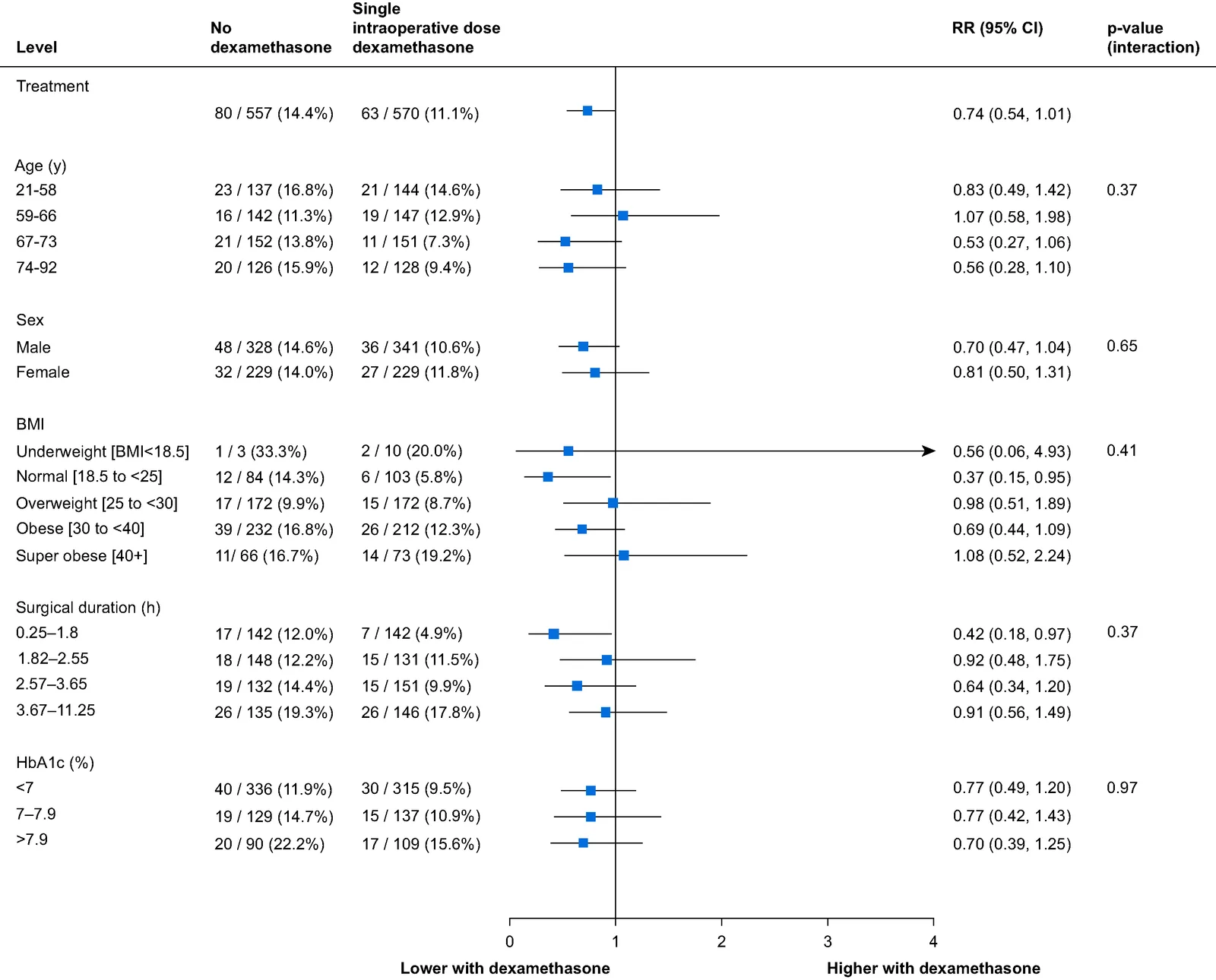
Subgroup analysis—surgical-site infection. The x-axis depicts relative risk. The widths of the confidence intervals for subgroup analyses were not adjusted for multiplicity and should not be used to infer definitive treatment effects.

As previously shown in the overall PADDI cohort,^5^ dexamethasone was not associated with an increased incidence of SSI in the patients with diabetes mellitus (dexamethasone 11.1% *vs* placebo 14.4%, RR 0.74 [95% CI 0.54–1.01], Fig. 4). The association of dexamethasone with SSI was consistent across all prespecified subgroups, including age, sex, BMI, surgical duration and HbA1c. In addition, after adjusting for confounders, there was no association between maximum perioperative blood glucose levels and SSI (RR 0.97 [95% CI 0.93–1.02], *P*=0.31), and no interaction between dexamethasone use and maximum perioperative glucose levels on SSI (RR 0.95 [95% CI 0.86–1.05], *P*=0.33). As shown in the PADDI study,^5^ there was no difference in length of hospital stay between dexamethasone and placebo groups (4.7 [2.9–6.9] *vs* 4.1 [2.8–6.3] days, *P*=0.30).

## Discussion

In this preplanned sub-study of the PADDI trial, we found that a single intraoperative dose of 8 mg of dexamethasone in participants with diabetes mellitus was associated with a maximum perioperative blood glucose 1.86 mM higher than placebo in the first 24 h, with a maximum effect after 8–12 h. Despite this, there were no increases in additional insulin usage, hypoglycaemic events or hospital length of stay. Furthermore, dexamethasone was not associated with increased SSI, and infection was not related to maximum perioperative blood glucose. These are important findings which have not been previously addressed in a large prospective, placebo-controlled, double-blinded study. They confirm that although glucose levels increase modestly for 24 h after intraoperative dexamethasone in participants with diabetes mellitus and HbA1c <9.0% undergoing non-urgent, noncardiac surgery, they are not associated with measurable adverse outcomes within the parameters studied.

Consistent with our findings, a number of systematic reviews have indicated that a single dose of intraoperative dexamethasone transiently increases blood glucose in people with diabetes mellitus. 13, 14, 15 These reviews, with differing inclusion criteria, showed that increases in blood glucose were 1.8–2.2 mM higher in patients receiving dexamethasone *vs* control (placebo or other antiemetic). The reviews included small randomised trials (largest sample size of 90, total number of patients <500) and larger retrospective cohort studies. Considerable heterogeneity was observed, with some retrospective studies showing no effect of dexamethasone on perioperative glucose levels. Reasons for heterogeneity include different dexamethasone doses and frequency, patient populations and data collection protocols. A more recent systematic review that only included randomised studies found that patients with diabetes mellitus (*n*=165) had a greater maximum increment in blood glucose with dexamethasone than those without diabetes mellitus (*n*=581) in the first 24 h (3.08 *vs* 0.29 mM).^16^

In our analysis, longer surgical duration was associated with a smaller difference in maximum perioperative blood glucose between dexamethasone and placebo. The effect appears to be because of higher maximum levels with longer operations in the placebo group, whereas there was no difference in the dexamethasone group. It is possible that the hyperglycaemic response is maximal after dexamethasone irrespective of surgical duration, whereas increased glucose in the placebo group with longer duration is because of a greater inflammatory response. Our finding contrasts with a small study of patients with and without diabetes mellitus that showed no association of surgical duration with maximum increase in glucose after surgery.^17^

As previously reported in the PADDI trial,^5^ we confirmed that dexamethasone was not associated with SSI incidence in patients with diabetes mellitus. This is consistent with a previous small randomised control trial,^18^ a number of retrospective cohort studies,^19^, ^20^, ^21^, ^22^ and systematic reviews and meta-analyses.^14^^,^^23^ In the overall PADDI cohort, patients with diabetes mellitus had a higher rate of SSI than those without (12.6% *vs* 8.0%, *P*<0.00001). Nevertheless, long- and short-term glycaemic control, as measured by HbA1c and maximum perioperative glucose, respectively, did not modulate the relationship between dexamethasone and SSI in the present study. Although the effect of dexamethasone on maximum perioperative glucose was modest, median maximum perioperative glucose levels in both dexamethasone and placebo groups exceeded the recommended maximum target of 10 mM. The transient effect of dexamethasone on hyperglycaemia during the first 24 h after surgery may have been insufficient to affect immune function in the context of contemporary management of hyperglycaemia and infection risk.

A major strength of the current study is that it is by far the largest prospective study to date, involving multiple sites across four countries and including multiple types of surgery. The parent PADDI trial was randomised, placebo-controlled, double-blind, and stratified by diabetes mellitus diagnosis. Only a small number of patients changed group in the current ‘as treated’ analysis, which was also adjusted for potential confounders. Further, intention-to-treat analysis of the diabetes mellitus stratum in the randomised study supported the glucose findings. These characteristics support the reliability and generalisability of the findings. The rigorous follow-up of SSI allows us to be confident of the validity of inferences relating to this result. Missing data are common in pragmatic trials, but there was a high compliance rate with only 0.2–5.1% missing values at each time point (Table S3), and the sensitivity analysis further underscores the validity of our findings.

However, there are a number of limitations. Patients with HbA1c >9% were excluded, so the findings do not apply to patients with very poor glycaemic control. In contrast, the findings are applicable to those with moderate control because 42.1% and 18.5% of participants had HbA1c ≥7.0% and >8.0%, respectively. Caution is recommended in extending the findings to patients with type 1 diabetes mellitus, as they represented <2% of participants. Glucose levels were only analysed in the first 24 h after surgery. However, previous studies showed that glucose levels after dexamethasone peak during this time.^13^ Given the pragmatic nature of the study, capillary and laboratory glucose levels were both included. However, levels measured by these methods were similar (Table S3), and a meta-analysis comparing laboratory and point-of-care testing in critically ill patients found an overall mean difference of <0.1 mM.^24^ Further, the time points of postoperative measurements were not precise, and the number of glucose measurements intraoperatively and in PACU was not specified. Nevertheless, there was a high compliance rate as stated above. Use of continuous glucose monitoring would provide additional information but was impractical in a study of this size. More specific details of baseline diabetes mellitus medications and insulin regimens and perioperative factors such as diet, fasting duration and intravenous fluids were not available. A future study comparing hyperglycaemic responses to dexamethasone in patients with and without diabetes mellitus would also be of interest.

In conclusion, this preplanned analysis of PADDI shows that a single intraoperative dose of dexamethasone was associated with a modest, transient increase in blood glucose perioperatively in patients with diabetes mellitus and HbA1c <9.0% undergoing non-urgent noncardiac surgery of at least intermediate severity. The increase was maximal 8 to 12 h after surgery, and no increase in SSI was observed. These observational findings suggest that the use of dexamethasone to prevent postoperative nausea and vomiting in patients with diabetes mellitus is safe with respect to SSI and other measured clinical outcomes despite the increased glycaemic response.

## Authors’ contributions

Study design, writing the manuscript: LAB, TBC, RJM, CM

Review, editing: TBC, RJM, PSM, EOL, DS, KH

Analysis, Review: CM, MAJ

Data acquisition, project administration: JS

## Funding

Australian National Health and Medical Research Council (ID 1079501); Research Grants Council of Hong Kong (GRF 14101816); Monash University.

The funders had no role in study design, data collection, data analysis, data interpretation, or writing of the report.

## Declaration of interest

The authors declare that they have no conflicts relevant to this manuscript.
