# Supplementary material for: The effect of intraoperative dexamethasone on glycaemic responses in people with diabetes mellitus: a preplanned analysis of the Perioperative ADministration of Dexamethasone and Infection trial
Source: Br J Anaesth. 2026 Jun 16;137(2):460–9. doi: 10.1016/j.bja.2026.05.009 (PMC13494095; doi:10.1016/j.bja.2026.05.009)
Supplement: Supplementary file 1 [file mmc1.docx]

**Supplementary material**

**PADDI investigators	Page 2**

**Table S1:** Baseline characteristics by dexamethasone status in patients with diabetes

**Page 4**

**Table S2:** Intention-to-treat analysis of the diabetes stratum of the original PADDI randomised population	**Page 6**

**Table S3:** Glucose levels above thresholds, test method and number of missing values	**Page 7**

**PADDI Chief investigators**

Tomás Corcoran, MD

Paul S. Myles, DSc,

Allen Cheng, PhD

Leon A. Bach, PhD

Kwok M. Ho, PhD

Matthew T.V.Chan, PhD

Kate Leslie, MD

Timothy G. Short, MD

Andrew Forbes, PhD

David Story, MD

Edmond O’Loughlin, M Clin Research

**PADDI site investigators and trial coordinators (with sites in alphabetical order)**

*Australia*

Alfred Hospital: P.S. Myles, S. Wallace, R. Han, A. Ditoro, M. Ueoka, A. Neylan

Austin Hospital: P. Peyton, G. Claxton, S. Baulch, S. Harris, S. Sidiropoulos

Ballarat Health Services: G. Henderson, K. Liyanapathirana, A. Mitchell, S. Whykes, L. Kolarik

Barwon Health: A. Iliov, A. Marriott, L. Brennan, M. Formica, R. Fiddes, E. Moore

Blacktown Hospital: A. Bullingham, J. Marshall, T. Sara, V. Wilkinson

Box Hill Hospital: S. Chew, L. Machado, A. Barton, J.S, Toh

Cabrini Health: A. Grossi, K. Barton

Canterbury Hospital: L. Ren, J. Kim, B. Correia, T. Krantis

Concord Repatriation General Hospital: R. Martin, B. Troy

Dandenong Hospital: R. Bulach, L. Bulfin, A. Wang, D. Bhatia-Dwivedi, T. Maggio

Epworth HealthCare Richmond: N. Tan, D. McCallum, A. Dalyell, V. Gordon

Fiona Stanley Hospital: E. O'Loughlin, M. Bailey, L. Glazov, A. Trehern, V. Finlay

Flinders Medical Centre: J. Koerber, A. Badenoch, S. Rattigan

Gold Coast University Hospital: C. Daniel, L. Sung

Gosford Hospital: B. Moran, A. Tandel, M. Keehan

John Hunter Hospital: A. Quail, R. Scott, J. Douglas

King Edward Memorial Hospital: N. McDonnell, D. Cavill

Mackay Base Hospital: D. Bartlett, G. Wright, A. Tait, T. Hess

Macquarie University Hospital: A. Chuan, L. Macdonald

Maroondah Hospital: R. Rengasamy, B. El-Behesy, A. Skiller, A. Wu

Monash Medical Centre: L. O’Halloran, D. Bhatia-Dwivedi, L. Bulfin

Peter MacCallum Cancer Centre: J. Hiller, B. Riedel, K. Yee, E. Crone, K. Coleman, S. McKeown

Prince of Wales Hospital NSW: M. Bennett, S. Cai, A. Holmewood, P. Marneros

Princess Alexandra Hospital: P. Sivalingam, D. Highton, A. Kearney, H. Hassan

Redcliffe Hospital: N. Bakri, M. Campher, M. Duroux, M. Ratcliffe

Rockingham Hospital: S. Hillyard, K. Bickell, S. Thomas

Royal Adelaide Hospital: T. Painter, J. Somfleth, C. Osborn, J. Hayer, L. de Prinse

Royal Brisbane and Women’s Hospital: J. Lee, H. Reynolds, M. Clarris, S. Holz, C. Town

Royal Darwin Hospital: B. Piper, M. Lyon, C. Gaylard

Royal Hobart Hospital: N. Terblanche, M. Challis, R, Seale, K. Button, S. Hannon

Royal Melbourne Hospital: K. Leslie, R. Cotter, C. Edgley, D. Middleton

Royal North Shore Hospital: B. Olesnicky, M. Doane, C. Player

Royal Perth Hospital: T. B. Corcoran, A. Toner, R. Way, S. March, P. Coutts

Royal Prince Alfred Hospital: T. McCulloch, G. Wong, K. Kramer, K. Connell

Sir Charles Gairdner Hospital: D. Ellyard, D. Currigan, P. Mickle, H. Jones

St John of God Hospital Ballarat: L.D. Paxton, C. Mitchell, R. Ray, D. McIntyre, V. Ward

St John of God Hospital Subiaco: T. B. Corcoran, Y. Buller, N. Hird

St Vincent’s Hospital Melbourne: T. Phan, J. Luxford, P. Corcoran, E. Fitzgerald, E. Gartner

Sunshine Coast University Hospital: N. Beauchamp, G. Godsall, S. Wadham, H. Rodgers

The Canberra Hospital: D. Nithyanandam, D. Babu

The Northern Hospital: A. Singh Nanuan, A. Singh Sarai

The Tweed Hospital: P. Correa, M. Campher

Wagga Wagga Rural Referral Centre: I. Lemech, C. Jenkins

Western Health: E. Hessian, D. Bramley, N. Sheridan, K. Gill, A. Tippett, S. Bates

Westmead Hospital: R. Halliwell, N. Phillips, F. Ali, L. Cope, E. Clark-Mackay, R. Paranthioene

Wollongong Hospital: N.A. Smith, F. Rouhani

*Hong Kong, China*

Prince of Wales Hospital, The Chinese University of Hong Kong: M.T.V. Chan, W.K.K. Wu, B. Fung, E. Lee, K. Hui

Tuen Mun Hospital: C. Lam, B. Cheng

*New Zealand*

Auckland City Hospital: M. Misur, T. Short, D. McAllister, S. Dandy, L. Ritchie

Auckland City Hospital CVICU: S. McGuinness, R. Parke, J. Dalton, M. Butler

Christchurch Hospital: R.R. Kennedy, M.A. McKellow

Hauora Tairawhiti Gisborne Hospital: R. Fuchs, R. Gray

Middlemore Hospital: F. Gilmour, S. Walker, H. Houston, S. Olliff

Manukau and Counties District Hospital: F. Gilmour, S. Walker, H. Houston, S. Olliff

*South Africa*

Groote Schuur Hospital: B. Biccard, M. Flint

**Table S1: Baseline characteristics by dexamethasone status in patients with diabetes**

|  | **Total** | **Placebo** | **Dexamethasone** | | | |
| --- | --- | --- | --- | --- | --- | --- |
|  | **N=1,130** | **N=560** | **N=570** | | | |
|  |  |  |  |  |  |  |
| **Ethnicity** |  |  |  | |  |  |
| **Caucasian** | 824 (72.9%) | 402 (71.8%) | 422 (74.0%) | |  |  |
| **Asian** | 205 (18.1%) | 102 (18.2%) | 103 (18.1%) | |  |  |
| **Other** | 101 (8.9%) | 56 (10.0%) | 45 (7.9%) | |  |  |
| **Country of study** |  |  |  | |  |  |
| **Australia** | 894 (79.1%) | 441 (78.8%) | 453 (79.5%) | |  |  |
| **Hong Kong** | 183 (16.2%) | 88 (15.7%) | 95 (16.7%) | |  |  |
| **New Zealand** | 52 (4.6%) | 30 (5.4%) | 22 (3.9%) | |  |  |
| **South Africa** | 1 (0.1%) | 1 (0.2%) | 0 (0.0%) | |  |  |
| **Chronic Pain – no. (%)** | 368 (32.6%) | 176 (31.4%) | 192 (33.7%) | | |  |
| **Opioids for chronic pain** | 125 (11.1%) | 58 (10.4%) | 67 (11.8%) | | |  |
| **Preoperative WHODAS score** | 16.0 (13.0-24.0) | 16.0 (13.0-24.0) | 16.0 (13.0-24.0) | | |  |
| **Type of surgery – no. (%)** |  |  |  | |  |  |
| **Gastrointestinal** | 324 (28.7%) | 147 (26.2%) | 177 (31.1%) | |  |  |
| **Gynaecology** | 94 (8.3%) | 53 (9.5%) | 41 (7.2%) | |  |  |
| **Neurosurgery** | 79 (7.0%) | 30 (5.4%) | 49 (8.6%) | |  |  |
| **Orthopaedics** | 197 (17.4%) | 105 (18.8%) | 92 (16.1%) | |  |  |
| **Urology-renal** | 196 (17.3%) | 106 (18.9%) | 90 (15.8%) | |  |  |
| **Vascular** | 80 (7.1%) | 42 (7.5%) | 38 (6.7%) | |  |  |
| **ENT, plastics + other** | 160 (14.2%) | 77 (13.8%) | 83 (14.6%) | |  |  |
| **Specific Procedures – no. (%)** |  |  |  | |  |  |
| **Breast surgery** | 22 (1.9%) | 12 (2.1%) | 10 (1.8%) | |  |  |
| **Spinal fusion** | 55 (4.9%) | 23 (4.1%) | 32 (5.6%) | |  |  |
| **Open reduction of fracture** | 8 (0.7%) | 5 (0.9%) | 3 (0.5%) | |  |  |
| **Herniorrhaphy** | 40 (3.5%) | 17 (3.0%) | 23 (4.0%) | |  |  |
| **Hip prosthesis** | 42 (3.7%) | 21 (3.8%) | 21 (3.7%) | |  |  |
| **Knee prosthesis** | 65 (5.8%) | 35 (6.2%) | 30 (5.3%) | |  |  |
| **Peripheral vascular bypass** | 34 (3.0%) | 16 (2.9%) | 18 (3.2%) | |  |  |
| **Re-fusion of spine** | 7 (0.6%) | 2 (0.4%) | 5 (0.9%) | |  |  |
| **None of the above** | 857 (75.8%) | 429 (76.6%) | 428 (75.1%) | |  |  |
| **Elective surgery** | 1,099 (97.3%) | 546 (97.5%) | 553 (97.0%) | |  |  |
| **Temperature at the time of wound closure (⸰C)** | 36.4 (36.0-36.9) | 36.4 (36.0-36.8) | 36.5 (36.0-36.9) | |  |  |
| **Total intravenous anesthesia – no. (%)** | 224 (19.8%) | 109 (19.5%) | 115 (20.2%) | |  |  |
| **Vapour-based anesthesia – no. (%)** | 943 (83.5%) | 469 (83.8%) | 474 (83.2%) | |  |  |
| **Nitrous Oxide therapy – no. (%)** | 75 (6.6%) | 39 (7.0%) | 36 (6.3%) | |  |  |
| **Muscle relaxant – no. (%)** | 1,063 (94.1%) | 527 (94.1%) | 536 (94.0%) | |  |  |
| **Opioid therapy – no. (%)** | 1,113 (98.5%) | 552 (98.6%) | 561 (98.4%) | |  |  |
| **Regional block – no. (%)** | 310 (27.4%) | 162 (28.9%) | 148 (26.0%) | |  |  |
| **Average intraoperative FiO_2_ (%)** | 0.5 (0.4, 0.6) | 0.5 (0.4, 0.5) | 0.5 (0.4, 0.6) | |  |  |

| **Table S2:** **Intention-to-treat analysis of the diabetes stratum of the original PADDI randomised population** | | | | |
| --- | --- | --- | --- | --- |
|  | **Placebo**  **(n=570)** | **Dexamethasone**  **(n=567)** | **Median difference**  **(95% CI)** | **p-value** |
| **Maximum perioperative blood glucose level (mmol L^-1^), median (IQR) ^*^** | 10.3 (8.8, 12.5) | 12.5 (10.6, 15.0) | 2.20 (1.76, 2.64) | <0.001 |

**^*^** Unadjusted analysis

| **Table S3: Glucose levels above thresholds, test method and number of missing values** | | | |
| --- | --- | --- | --- |
|  | **Total** | **No Dexamethasone** | **Dexamethasone** |
|  | N=1,130 | N=560 | N=570 |
| **Blood glucose on induction (mmol L^-1^), median (IQR)** | **6.9 (5.8-8.3)** | **6.8 (5.7-8.2)** | **7.0 (5.9-8.5)** |
| Missing | 43 (3.8%) | 18 (3.2%) | 25 (4.4%) |
| ≥ 10 mmol L^-1^ | 129 (11.9%) | 59 (10.9%) | 70 (12.8%) |
| ≥ 12 mmol L^-1^ | 49 (4.5%) | 23 (4.2%) | 26 (4.8%) |
| ≥ 15 mmol L^-1^ | 9 (0.8%) | 6 (1.1%) | 3 (0.6%) |
| Test method |  |  |  |
| Laboratory, n (%) | 153 (14.1%) | 76 (14.0%) | 77 (14.1%) |
| POC*, n (%) | 934 (85.9%) | 466 (86.0%) | 468 (85.9%) |
| Laboratory, mmol L^-1^ | 7.2 (6.2-8.7) | 7.2 (6.1-8.5) | 7.3 (6.2-8.9) |
| POC, mmol L^-1^ | 6.8 (5.8-8.3) | 6.8 (5.7-8.2) | 6.8 (5.9-8.4) |
| **Maximum intraoperative blood glucose, median (IQR)** | **8.9 (7.0-10.6)** | **8.2 (6.8-10.2)** | **9.4 (7.5-11.3)** |
| Missing | 48 (4.2%) | 19 (3.4%) | 29 (5.1%) |
| ≥ 10 mmol L^-1^ | 354 (32.7%) | 144 (26.6%) | 210 (38.8%) |
| ≥ 12 mmol L^-1^ | 158 (14.6%) | 55 (10.2%) | 103 (19.0%) |
| ≥ 15 mmol L^-1^ | 35 (3.2%) | 13 (2.4%) | 22 (4.1%) |
| Test method |  |  |  |
| Laboratory, n (%) | 169 (15.6%) | 90 (16.6%) | 79 (14.6%) |
| POC, n (%) | 913 (84.4%) | 451 (83.4%) | 462 (85.4%) |
| Laboratory, mmol L^-1^ | 8.8 (7.3-10.8) | 8.2 (6.7-10.4) | 9.7 (8.2-12.0) |
| POC, mmol L^-1^ | 8.9 (7.0-10.6) | 8.2 (6.8-10.1) | 9.4 (7.5-11.2) |
| **Maximum blood glucose in PACU, median (IQR)** | **9.4 (7.8-11.3)** | **8.7 (7.1-10.4)** | **10.2 (8.6-12.1)** |
| Missing | 5 (0.4%) | 4 (0.7%) | 1 (0.2%) |
| ≥ 10 mmol L^-1^ | 465 (41.3%) | 165 (29.7%) | 300 (52.7%) |
| ≥ 12 mmol L^-1^ | 213 (18.9%) | 63 (11.3%) | 150 (26.4%) |
| ≥ 15 mmol L^-1^ | 44 ( 3.9%) | 9 ( 1.6%) | 35 ( 6.2%) |
| Test method |  |  |  |
| Laboratory, n (%) | 68 (6.0%) | 29 (5.2%) | 39 (6.9%) |
| POC, n (%) | 1,056 (94.0%) | 527 (94.8%) | 529 (93.1%) |
| Laboratory, mmol L^-1^ | 9.9 (8.1-11.3) | 9.3 (7.4-10.6) | 10.1 (9.1-12.4) |
| POC, mmol L^-1^ | 9.4 (7.8-11.3) | 8.6 (7.1-10.4) | 10.2 (8.6-12.1) |
| **Blood glucose (POC) 8 – 12 hours, median (IQR)** | **10.1 (8.3-12.7)** | **9.0 (7.6-10.9)** | **11.4 (9.3-14.1)** |
| Missing | 11 (1.0%) | 5 (0.9%) | 6 (1.1%) |
| ≥ 10 mmol L^-1^ | 566 (50.6%) | 186 (33.5%) | 380 (67.4%) |
| ≥ 12 mmol L^-1^ | 337 (30.1%) | 95 (17.1%) | 242 (42.9%) |
| ≥ 15 mmol L^-1^ | 130 (11.6%) | 30 (5.4%) | 100 (17.7%) |
| **Blood glucose day 1, median (IQR)** | **8.8 (7.3-11.0)** | **8.4 (6.9-10.3)** | **9.3 (7.7-11.7)** |
| Missing | 8 (0.7%) | 2 (0.4%) | 6 (1.1%) |
| ≥ 10 mmol L^-1^ | 373 (33.2%) | 149 (26.7%) | 224 (39.7%) |
| ≥ 12 mmol L^-1^ | 198 (17.6%) | 74 (13.3%) | 124 (22.0%) |
| ≥ 15 mmol L^-1^ | 71 (6.3%) | 24 (4.3%) | 47 (8.3%) |
| **Blood glucose by test method** |  |  |  |
| Laboratory, n (%) | 347 (30.9%) | 163 (29.2%) | 184 (32.6%) |
| POC, n (%) | 775 (69.1%) | 395 (70.8%) | 380 (67.4%) |
| Laboratory, mmol L^-1^ | 8.8 (7.4-10.9) | 8.6 (7.2-10.7) | 9.1 (7.6-11.7) |
| POC, mmol L^-1^ | 8.8 (7.3-11.1) | 8.3 (6.9-10.2) | 9.4 (7.7-11.7) |

* POC, point of care
